# Urinary metabolite signatures as predictive biomarkers for estrus detection in water buffaloes: a proton-NMR based study

**DOI:** 10.1080/01652176.2025.2593361

**Published:** 2025-12-13

**Authors:** Suman Sangwan, M. H. Jan, Ekta Hooda, Renu Choudhary, Sunesh Balhara, Rupali Rautela, Sarita Yadav, S. K. Phulia, R. K. Sharma, Vijay Paul, Yash Pal, Mehar Singh Khatkar, Ashok Kumar Balhara

**Affiliations:** a ICAR-Central Institute for Research on Buffaloes, Hisar, India; b Department of Veterinary Biochemistry, LUVAS, Hisar, India; c ICAR-National Research Centre on Yak, Dirang, India; d Davies Livestock Research Centre, SAVS, The University of Adelaide, Adelaide, Australia

**Keywords:** Urine, metabolites, estrus cycle, NMR, buffalo

## Abstract

Developing a reliable, field ready estrus detection method is crucial for improving buffalo reproduction due to their subtle and poorly expressed estrus signs. This study investigated estrus phase-specific urinary metabolites in cyclic Murrah buffaloes (n=6) using proton nuclear magnetic resonance (^1^H-NMR). A total of 90 urinary metabolites were identified, with 15 consistently detected across all animals during the estrus phases (proestrus, estrus and diestrus). PCA highlighted p-cresol, ornithine, phenol, chlorogenate, quinolinate, hippurate and 2-hydroxyisobutyrate as key metabolites differentiating the estrus phases. PLS-DA identified p-cresol and phenol for estrus; chlorogenate and o-acetylcholine for proestrus and ornithine in diestrus as the potential urinary markers for detection of estrus phases based on their VIP scores greater than 1.5. Metabolic pathway analysis revealed that the glycerophospholipid pathway, phenylalanine, tyrosine and tryptophan biosynthesis, aspartate and aldarate metabolism, and starch and sucrose metabolism were the major metabolic pathways involved in regulating and controlling estrus cycle. Notably, p-cresol and phenol exhibited significant abundance during estrus (over 9-fold and 5-fold, respectively), suggesting their potential as putative estrus detection biomarkers. However, given the limited sample size (*n* = 6), these findings should be considered preliminary, and independent validation in larger, well-characterized cohorts is needed to confirm diagnostic utility.

## Graphical abstract

**Figure:**
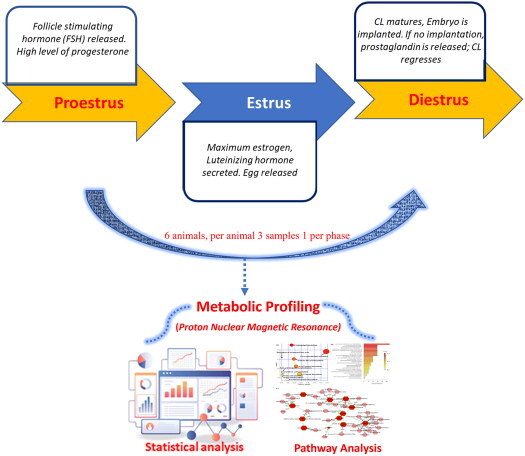

## Introduction

The estrus cycle, a cascade of physiological events regulated by the hypothalamic-pituitary-gonadal (HPG) axis, governs reproductive function and fertility in female mammals (Schalich et al. 2024). In buffaloes, the cycle repeats every 19–21 days, with a short window (12–30 h) of sexual receptivity (estrus phase). The estrus cycle involves hormonal fluctuations that drive metabolic changes at cellular level, supporting follicular development, ovulation, and subsequent reproductive events (Abdulkareem 2012, 2013). Metabolites generated during the process are not just the substrates or products of metabolic events but also regulates cell phenotype and behavior (Baker and Rutter 2023). Understanding the dynamic levels of these metabolites across estrus cycle is essential for unraveling the underlying mechanisms regulating reproductive physiology and health in female animals.

Omics-approaches measure global expressions of proteins, lipids, RNAs, and metabolites, providing a snapshot of biological processes (Marrella and Biase 2023; Casaro et al. 2025). Among these, metabolomics (untargeted profiling of metabolites) has emerged as a powerful platform to identify metabolite signatures linked to physiological and pathological states (Johnson et al. 2016). Metabolomics has also been applied to investigate the metabolic changes during estrus cycle, pregnancy and other reproductive events in various mammalian species (Du et al. 2023; Hessock et al. 2023; Shi et al. 2023; Doshi et al. 2024; Ren et al. 2024; Zhao et al. 2024). Multi-omics approaches have revealed metabolic dysregulation linked to subfertility in heifers (Marrella and Biase 2023). A distinct vaginal fluid metabolome (including pheromone-like molecules), was identified across murine estrus cycle phases (Matějková et al. 2024). Additionally, 2-Pentanone was proposed as a biomarker for estrus in cattle cervicovaginal mucus (Pluta et al. 2021).

Urine is a preferred biological sample for metabolic analysis due to its rich metabolite content and non-invasive collection method, which minimizes animal stress (Bouatra et al. 2013). Studies across species, including cattle and yak, have demonstrated significant changes in urinary metabolites during the estrus cycle and early pregnancy (Doshi et al. 2024; Ren et al. 2024; Tang et al. 2024; Zhao et al. 2024). In buffalo, previous NMR-based studies have primarily focused on pregnancy related biomarkers (Sarangi et al. 2022), but urine based ^1^H-NMR has not yet been applied to estrus detection. This reveals a clear methodological gap that the present study addresses by characterizing phase-specific urinary metabolite profiles using NMR. Metabolite exploration is commonly done by nuclear magnetic resonance (NMR), and mass spectrometry (MS), often coupled with separation techniques such as liquid chromatography (LC), gas chromatography (GC) and supercritical fluid chromatography (SFC) (Rakusanova and Cajka 2024).

Buffaloes are vital contributors to India’s dairy sector, accounting for about 50% of total milk production (DAHD, 2024). However, their suboptimal reproductive efficiency – driven by poor behavioral estrus expression and short estrus duration (Gautam et al. 2024) hampers breeding success. In our recent study,^1^H NMR-based untargeted metabolomics revealed twenty-four differentially expressed metabolites during pregnancy in buffaloes and mithuns, with anthranilate, 3-hydroxykynurenine, and tyrosine identified as key metabolites (Sarangi et al. 2022; Sangwan et al. 2024). However, among these, detailed information on urinary metabolites across distinct phases of the estrus cycle in buffalo remains limited, highlighting a critical research gap. To address this, metabolic profiling of urinary biomarkers offers a strategy to decode phase-specific metabolic signatures of estrus cycle. We hypothesize that specific urinary metabolites vary predicably across proestrus, estrus, and diestrus and can serve estrus biomarkers in Murrah buffaloes. This study employs proton NMR-based metabolomics to identify key urinary metabolites across estrus phases, aiming to improve estrus detection and informing buffalo breeding protocols.

## Materials and methods

### Experimental animals

The study was conducted on eighteen Murrah (multiparous, cyclic) buffaloes, aged 4 to 8 years, with a body condition score of 3.0 to 3.5 on a 0-to-5-point scale, selected from the organized herd at the animal farm of ICAR - Central Institute for Research on Buffaloes (ICAR-CIRB) in Hisar, India. The buffaloes had a mean body weight of 436 ± 52 kg (mean ± SE). Throughout the study, the experimental animals were housed under semi-intensive conditions and managed according to standardized managemental practices. The feeding regimen adhered strictly to the guidelines set by the Indian Council of Agricultural Research (ICAR, 2013), which prescribed a daily dry matter intake equivalent to 2.5% of the animal’s body weight. Roughage included fresh berseem (Trifolium Alexandrium) and wheat straw provided *ad libitum*. A farm–standard concentrate (formulated to ∼ 16–18% crude protein and ∼ 65–70% total digestible nutrients) comprised ground wheat, maize, barley, rice bran, mustard cake, 2% mineral mixture and 1% common salt. Additionally, all buffaloes were provided *ad libitum* access to drinking water to ensure their hydration needs.

### Estrus detection

The buffaloes in estrus were identified by parading of a teaser bull, and observing behavioral estrus signs, along with clinical examination of the genital tract by transrectal palpation. Additionally, cyclic animals in estrus were confirmed by the absence of corpus luteum (CL) and presence of a large dominant ovulatory follicle (>12 mm size) on one of the ovaries, as determined by transrectal ultrasonography examination using a ‘B-mode’ ultrasound scanner (Hitachi Aloka F31, Japan) equipped with an intraoperative convex 10–4 MHz probe (UST-995-7.5). These finding were complemented by presence of good uterine tonicity and cervical discharge observed during per rectum palpation.

All eighteen animals were thoroughly screened before being included in the urine sampling plan. No urine was collected in the first observed estrus. For the actual NMR studies, urine samples were collected from six cyclic buffaloes that met the above-described criteria, out of the total eighteen buffaloes initially introduced in the study. Urine samples for NMR studies were collected from these six cyclic buffaloes during three different phases of their subsequent estrus cycle namely estrus (0 day), diestrus (10–12 days) and proestrus (18–20 days).

### Urine sample collection and preservation

Naturally voided mid-stream urine samples (approximately 250 mL) were collected in wide-mouth collection jars from each cyclic buffaloes (*n* = 6) in the morning (around 0700 hrs) on days 0 (day of estrus), 10–12 (diestrus), and 18–20 (proestrus). Each urine sample was filtered through a nylon syringe filter (0.45 μm pore size, 25 mm diameter, Axiva Sichem Pvt. Ltd., Haryana, India), and the filtrate was collected into a 15 mL centrifuge tube. The urine was then mixed with diethyl ether in a 1:1 ratio and shaken vigorously to allow the volatile metabolites to partition into the ether phase (Drabińska et al. 2020). The ether phase, containing the volatile metabolites was stored at −80 °C for further NMR analysis.

### ^1^H NMR spectroscopy analysis

Urine samples were reconstituted with 400 μL 0.2 M sodium phosphate buffer (pH 7.0), centrifuged at 14,000 × g at 4 °C for 10 mins. For each sample, 400 μL of resulting supernatant was mixed with 230 μL of NMR buffer (0.2% w/v) containing sodium azide (NaN_3_) 0.2 M sodium phosphate buffer (Na_2_HPO_4_NaH_2_PO_4_) and 60 μl of TSP stock solution (1 mg/ml sodium 3-trimethylsilyl-(2, 2, 3, 3-D4) propionate in CDCl_3_. The mixture was vortexed for 30 s to ensure homogeneity and to achieve a final pH of 7.4 (Bertram et al., 2011). The prepared sample was transferred to 5 mm NMR tube for *^1^H* NMR spectral analysis.

*^1^H-NMR* spectra was acquired on a Bruker Avance 400 spectrometer (400.11 MHz, 298 K; Bruker Biospin, Rheinstetten, Germany) using a 1D pulse sequence with water pre-saturation (recycle delay: 1s; mixing time: 100 ms; 90° pulse: 13.46 μs). Free induction decays (FIDs) were collected over 16 scans (65,536 points, spectral width: 8012.82 H, acquisition time: 4.08 s), processed exponential line broadening (0.3 Hz), zero-filled by factor of two, and Fourier transformed. Spectra were phase and baseline corrected and referenced to TSP (δ 0.00) using TopSpin 3.2 (Bruker Biospin, Germany).

Spectra (δ 10 − 0.5) were processed using Mestre Nova 6.0.2-5475 (Mestrelab Research, Spain) for referencing, phase, and baseline correction. Metabolite identification and quantification was performed using Chenomx NMR suite 9.0 (Chenomx Inc, Canada), referencing TSP at 0 ppm. The Livestock Metabolite Database (http://www.lmdb.ca), Bovine Metabolite Database (http://www.bmdb.ca), and Chenomx library were utilized for metabolite identification.

### Statistical and metabolic pathway analysis

Metabolite data were analyzed using R (version 4.4.0; R Core Team, 2024) and MetaboAnalyst 6.0 (http://www.metaboanalyst.ca) for statistical and pathway analysis. Metabolite profiles were initially assessed using one-way ANOVA, with individual metabolites tested for significant differences across the three estrus stages; metabolites demonstrating statistically significant variations (*p* < 0.05) were subsequently selected for further analysis. The metabolite data were normalized using sum normalization and mean centering. Residuals were assessed for normality (Shapiro-Wilk test) and homogeneity of variance, supporting the use of ANOVA for between-phase comparisons. Dimensionality reduction was performed using Principal Component Analysis (PCA), robust Principal Component Analysis (rPCA), and Partial Least Squares Discriminant Analysis (PLS-DA). The PLS-DA optimized class discrimination among estrus, proestrus, and diestrus by maximizing covariance between predictors and class labels, and Variable Importance in Projection (VIP) scores were used to identify key metabolites. To minimize overfitting, models were validated by five-fold cross-validation and 100 permutation testing, with permuted R^2^ and Q^2^ values lower than the original model (*p* < 0.01). Pathways analysis utilized a *Bos taurus* library and Kyoto Encyclopedia of Genes and Genomes (KEGG; http://www.kegg.com) databases, comparing metabolites in biofluids and other species.

### Ethical approval statement

The experiments conducted for this study were approved by the Institute Animal Ethics Committee (IAEC) (Registration no: 267/GO/RBi/L/2000/CPCSEA) of the Central Institute for Research on Buffaloes (IAEC-CIRB/23-24/A/004 dated Aug 28, 2023), under the supervision of the Committee for Control and Supervision of Experiments on Animals (CCSEA), a statutory committee of the Department of Animal Husbandry and Dairying, Ministry of Fisheries, Animal Husbandry and Dairying, Government of India, established under the Prevention of Cruelty to Animals (PCA) Act, 1960. All animals used in the experiment were maintained under scientific conditions and experienced no additional discomfort during sample collection for this study.

## Results

### Identification of the differentially expressed urinary metabolites

A total of ninety metabolites were identified through ^1^H NMR analysis in the urine samples of buffaloes during three phases of estrus (estrus, proestrus, and diestrus) cycle. Among these, fifteen metabolites were consistently detected in all animals on relevant collection days and showed significant differences in their levels across the estrus phases (estrus, proestrus, and diestrus) in buffalo urine (Table 1). The levels of five metabolites: p-Cresol, Phenol, 2-Oxovalerate, 2-Hydroxyisobutyrate and Glucose were distinctly higher (*p* < 0.05) at the estrus as compared to proestrus and diestrus phases. However, Chlorogenate, Glycine, Glycocholate, Ascorbate and 3-Hydroxykynurenine were significantly higher during proestrus phase in buffalo. Whereas, the levels of p-Cresol, Phenol, o-acetylcholine, Glucuronate, 3-hydroxykynurenine and 3-indoxylsulfate were significantly low at diestrus phase. Among the significantly altered metabolites, p-cresol and phenol emerged as the strongest estrus-associated markers, with very large effects and extreme fold changes across phases. For p-cresol, ANOVA indicated a robust phase effect, F (2,10) = 463.77, *p* = 3.26 × 10^–14^, FDR = 1.22 × 10^–13^, *η*_p_^2^ = 0.989; concentrations were 5.3-fold higher in estrus than proestrus and nearly 3900-fold higher than post estrus.

**Table 1.** Urinary concentrations of metabolites across the phases of estrus cycle phases (in milli/microMol) in Murrah buffaloes (mean ± SD).

Metabolite	Estrus	Diestrus	Proestrus
p-Cresol	112.35 ± 4.86^a^	2.49 ± 0.57^c^	12.04 ± 1.20^b^
Phenol	77.64 ± 4.03^a^	0.02 ± 0.02^c^	14.80 ± 2.78^b^
Chlorogenate	0.23 ± 0.05^b^	0.21 ± 0.08^b^	10.41 ± 4.94^a^
o-acetylcholine	16.52 ± 1.44^a^	0.31 ± 0.10^b^	12.57 ± 0.41^a^
Glycine	0.07 ± 0.02^b^	0.47 ± 0.12^b^	6.27 ± 0.49^a^
Ornithine	16.11 ± 3.59^a^	17.70 ± 2.55^a^	11.58 ± 0.82^a^
Glucuronate	4.12 ± 0.16^a^	0.72 ± 0.07^b^	6.32 ± 0.55^a^
Glycocholate	1.63 ± 0.68^b^	0.01 ± 0.00^b^	4.89 ± 0.51^a^
Ascorbate	0.01 ± 0.00^b^	0.25 ± 0.07^b^	3.81 ± 0.60^a^
2-Oxovalerate	10.32 ± 0.90^a^	0.16 ± 0.09^b^	0.22 ± 0.11^b^
2-Hydroxyisobutyrate	13.91 ± 0.97^a^	2.18 ± 0.59^b^	1.64 ± 0.56^b^
Glucose	15.83 ± 1.04^a^	4.32 ± 0.63^b^	2.44 ± 0.43^c^
5-Hydroxytryptophan	0.15 ± 0.04^b^	0.49 ± 0.04^b^	3.29 ± 0.59^a^
3-hydroxykynurenine	1.61 ± 0.49^b^	0.53 ± 0.15^c^	3.75 ± 0.79^a^
3-indoxylsulfate	4.54 ± 0.42^a^	0.26 ± 0.12^b^	4.72 ± 0.99^a^

Mean with different superscripts (a, b and c) within the row i.e. between different phases of estrus differ significantly (*p* < 0.05). ‘a’ denotes the highest mean concentration, followed by ‘b’ and ‘c’ in descending order.

### Multivariate analysis of the urine metabolites

**Figure 1.**
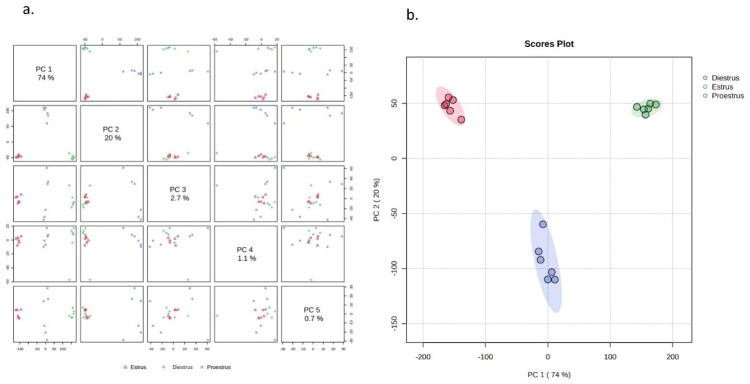
(a). Principal components analysis (PCA) plot elucidating the variability (PC1 74%, PC2 20%, PC3 2.7%, PC4 1.1% and PC5 0.7%) of metabolomic data. (b) Scores plot showing distinct clustering of buffalo urine metabolomic profiles during different estrus phases. Each point represents an individual sample, with colored ellipses indicating 95% confidence regions for each estrus phase.

Principal Component Analysis (PCA) was employed using five principal components, PC1 (74%), PC2 (20%), PC3 (2.7%), PC4 (1.1%), and PC5 (0.7%) to discern intrinsic metabolomic variation patterns in urine across three phases of the estrus cycle in buffalo. The PCA plots (Figure 1a) depicted separation of estrus (red scores), diestrus (green scores), and proestrus (blue scores) samples with around 94% variability in the metabolite profiles explained by PC1 and PC2 combined. Moreover, the first three principal components collectively explained 96.7% of the dataset’s variability.

The PCA 2-D score plot as depicted (Figure 1b) reaffirmed the distinct clustering across the estrus, diestrus, and proestrus phases, whereas, biplot (Supplementary Figure 1) for PCA and rPCA (PC1 and PC2) further clearly identified the key dimensions and metabolites (p-cresol, phenol, ornithine, chlorogenate, o-acetylcholine) explaining variability. The PCA loadings recognized p-cresol, ornithine, phenol, chlorogenate, o-acetylcholine, hippurate and glycine as the prominent metabolites responsible for differentiation among the different phases of estrus in buffalo urine (Supplementary Figure 2). To statistically validate the differences observed between the three phases, PERMANOVA was performed. The overall analysis indicated significant differences between the groups (*F* = 34.99, R^2^ = 0.824, p-value = 0.002). Pairwise comparisons showed clear separations between all phases (estrus vs diestrus: *F* = 35.04, R^2^ = 0.778; estrus vs proestrus: *F* = 53.62, R^2^ = 0.843; proestrus vs diestrus: *F* = 21.36, R^2^ = 0.681; Table 2).

**Table 2.** Pairwise PERMANOVA results showing differences between estrus phases using buffalo urine metabolomic profiles.

Comparison	F-value	R²	PERMANOVA *p*-value	Dispersion
Overall	35	0.824	0.0020	0.029
Estrus vs diestrus	35.04	0.778	0.0312	0.094
Estrus vs proestrus	53.62	0.843	0.0312	0.021
Diestrus vs proestrus	21.36	0.681	0.0312	0.307

Partial Least Squares Discriminant Analysis (PLS-DA) applied on the dataset further confirmed group separation and corresponding differential metabolites profile. A clear separation of groups (estrus, diestrus, and proestrus phases of estrus) as illustrated in Supplementary Figure 3 indicated that PLS-DA captured the variation associated with the different estrus phases, emphasizing the discriminatory power of these components.

**Figure 2.**
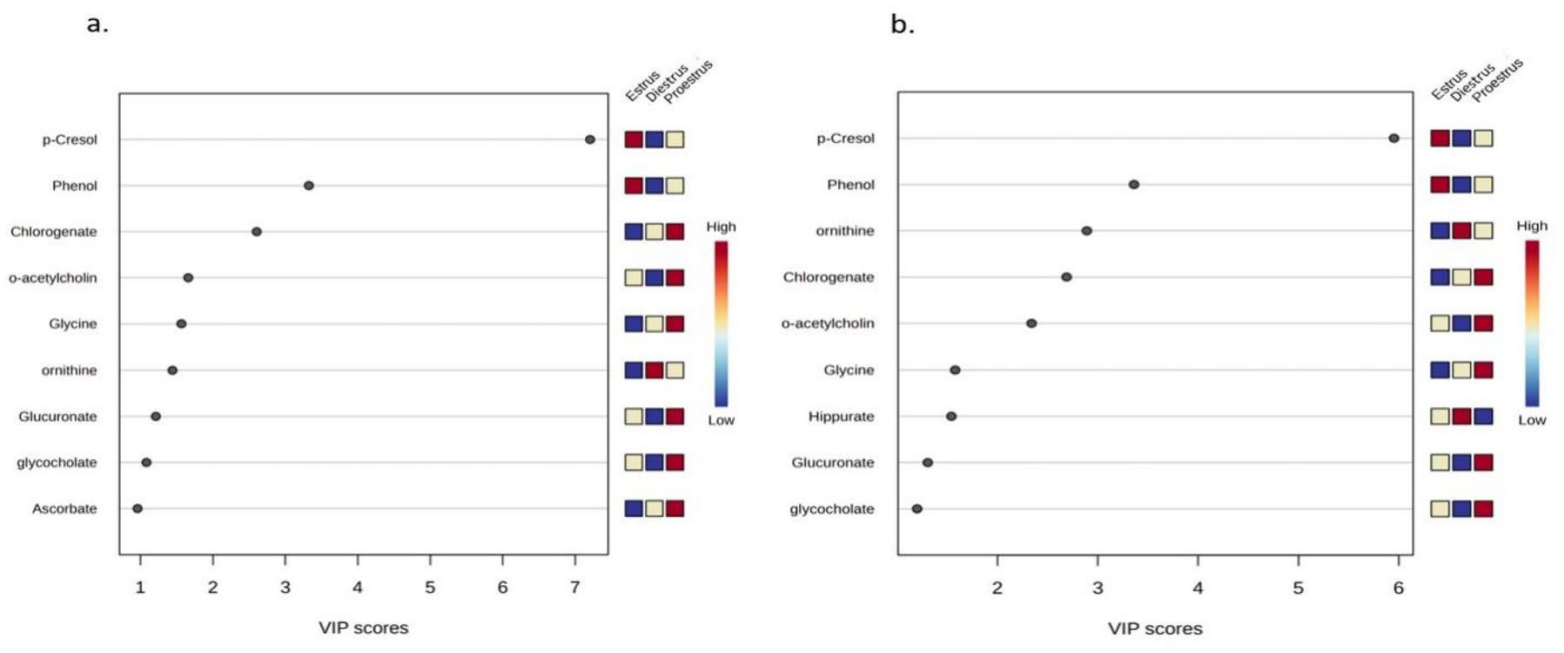
Variable Importance in projection (VIP) scores of the metabolites analyzed through PLS-DA for PC1 (left) and PC2 (right); the abundance ratio (corresponding heat-map) depicting p-cresol and phenol for estrus, chlorogenate and o-acetylcholine for pro-estrus and ornithine for diestrus as the potential urinary markers in buffalo.

Variable Importance in Projection (VIP) scores were obtained to evaluate variables’ importance in the PLS-DA model and were calculated as a weighted sum of the squared correlations between the PLS-DA components and the original variable. The weights correspond to the variation explained by the PLS-DA component, with higher VIP scores indicating greater importance in differentiating groups. The metabolites with VIP scores above 1.2 (Figure 2) were identified as potential urinary markers for estrus phases: p-cresol (7.206) and phenol (3.325) for estrus, chlorogenate (2.605), o-acetylcholine (2.340) and glycine (1.578) for proestrus and ornithine (2.891) in diestrus estrus cycle.

**Figure 3.**
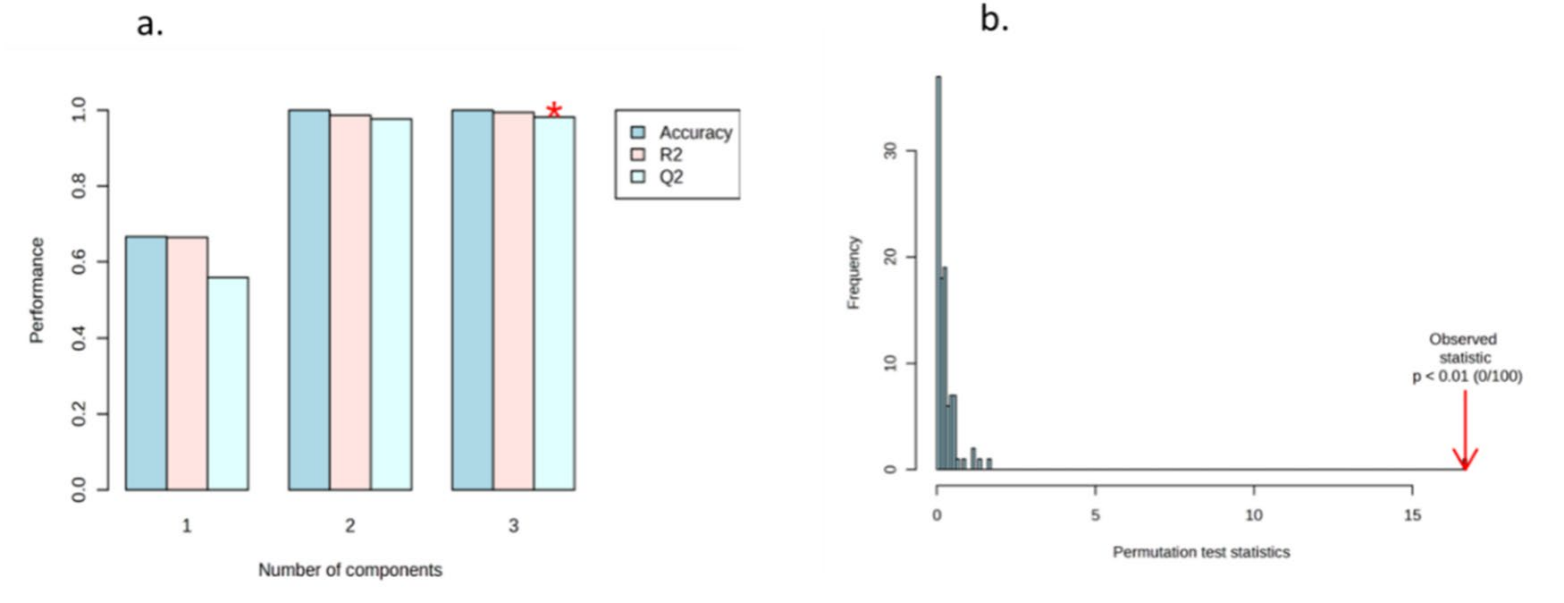
(a) The cross-validation test PLS-DA model showing cumulative values of R2 = 99.45% and Q2 = 98.13%, and demonstrating a good distinction between the three phases of estrus. (b) Century of permutations showing significant results (*p* < 0.01).

The performance of the PLS-DA model was evaluated through five-fold cross-validation using Q^2^ and R^2^ values. Conventionally, a PLS-DA model is considered a good fit if R^2^ or Q^2^ values exceed 70%. Cross-validation showed cumulative R^2^ of 99.45% and Q^2^ of 98.13%, with score plots illustrating clear clustering (Figure 3a), and permutation testing (*n* = 100) confirming statistical significance (Figure 3b). Given the small sample size (*n* = 6), these very high R^2^/Q^2^ values may partly reflect overfitting; therefore, they should be interpreted with caution, and external validation on larger, independent cohorts will be essential to confirm robustness.

**Figure 4.**
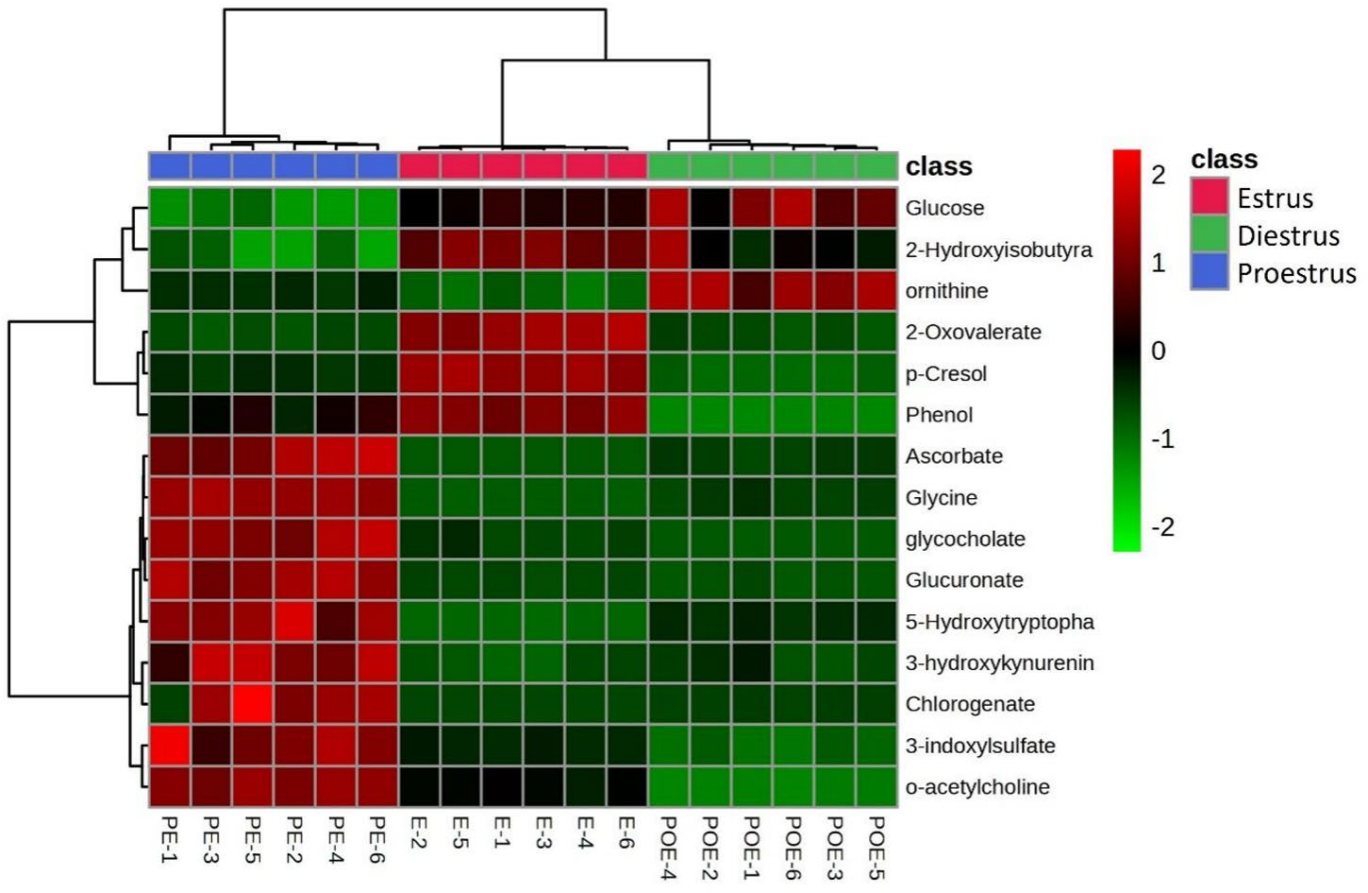
The heatmap showing the abundance of differentially expressed metabolites and their hierarchical clustering on various phases of estrus cycle.

The hierarchical clustering of the differentially expressed metabolites depicted in the heat map also revealed intriguing patterns, with metabolites grouping by phase specific expression profiles; phase-specific samples clustered into separate branches within the dendrogram (Figure 4).

### Pathway analysis

**Figure 5.**
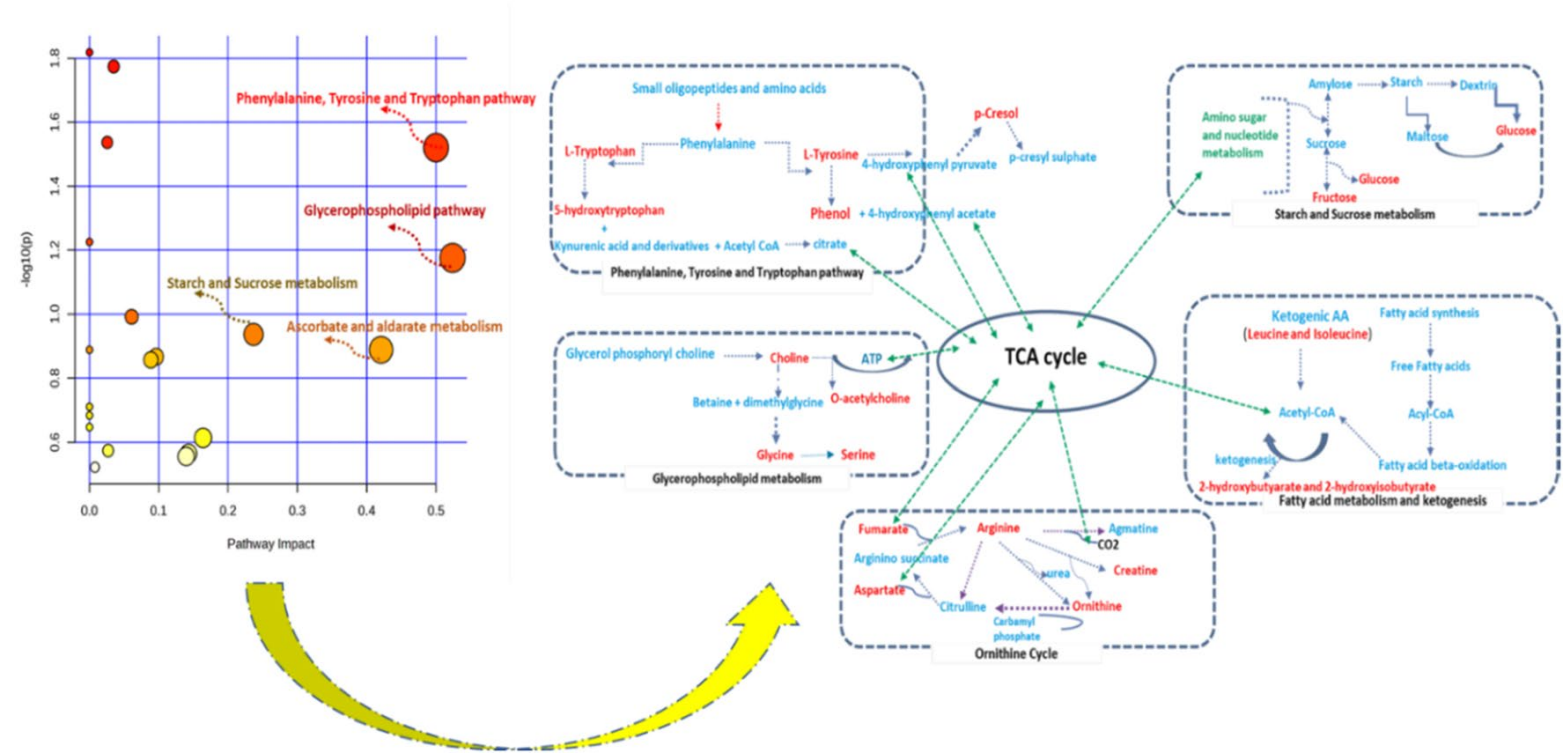
Bubble plots depicting the important metabolic pathways (glycerophospholipid pathway, phenylalanine, tyrosine and tryptophan biosynthesis, Ascorbate and aldarate metabolism, and starch and sucrose metabolism) and their impact on the estrus cycle with MetaboAnalyst 6.0.

To interpret the functional significance of differentially expressed urinary metabolites across various phases of the estrus cycle in buffaloes, MetaboAnalyst 6.0 was used to identify the associated metabolic pathways. The input for this analysis was the dataset of differentially expressed urinary metabolites obtained during the pro-estrus, estrus, and diestrus phases. Pathway topology analysis revealed that these metabolites are primarily involved in regulating glycometabolism, amino acid metabolism, and lipid metabolism through four key pathways with an impact value above 0.2: (1) Glycerophospholipid metabolism, (2) Phenylalanine, tyrosine, and tryptophan biosynthesis, (3) Ascorbate and aldarate metabolism, and (4) Starch and sucrose metabolism (Figure 5). Additionally, the analysis identified several key metabolites within these pathways that play significant roles in regulating and modulating the metabolic processes associated with different phases of the estrus cycle in buffaloes.

**Figure 6.**
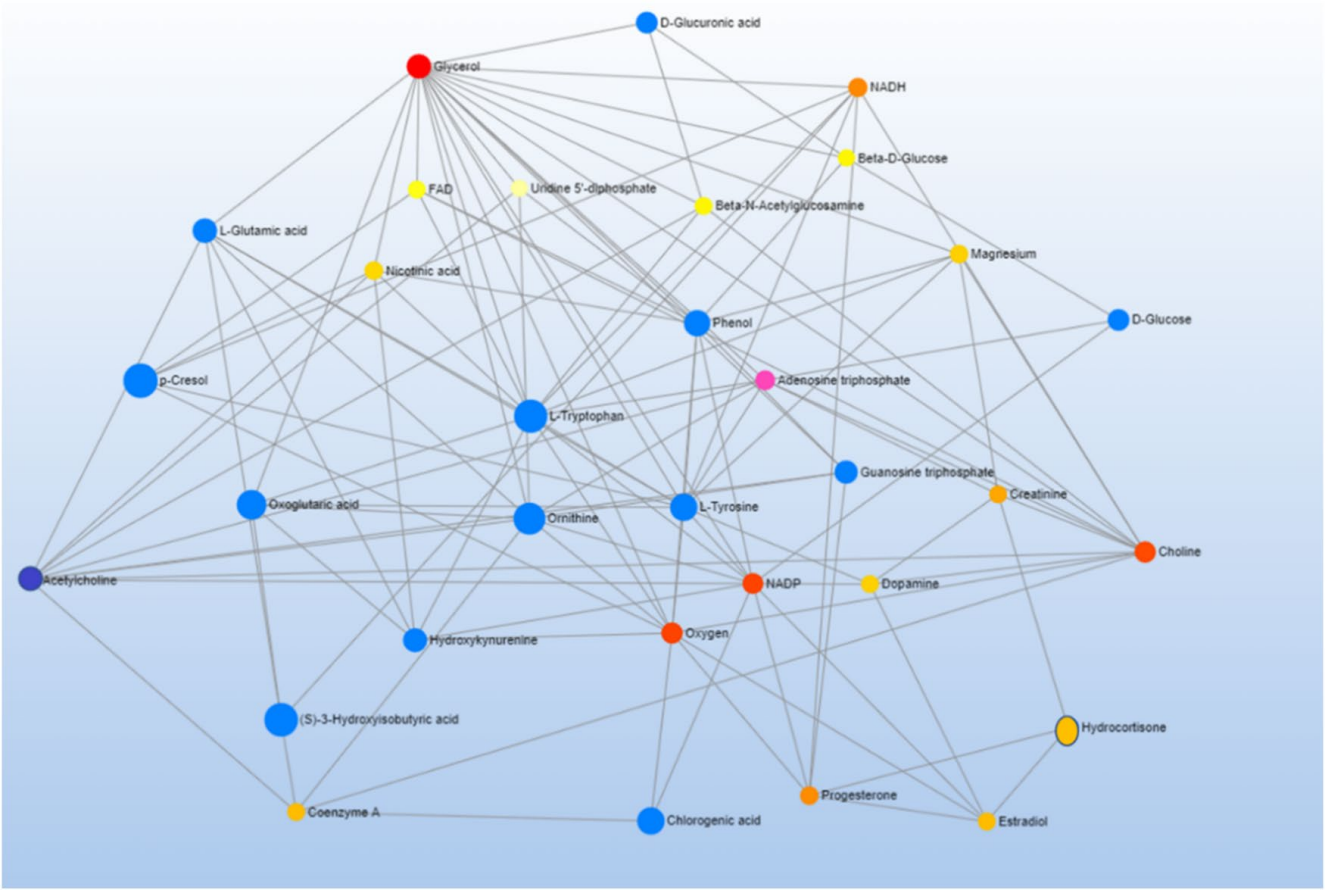
MetaboAnalyst 6.0 Network connection analysis showing the cross and interlinks of metabolites with each other, hormones (progesterone, estradiol and hydrocortisone) and with energy units, co-enzymes & metallic metabolites (NADP, NADPH, FAD, oxygen, magnesium and coenzymes). The blue dots represent the metabolites found in our study.

Further, Figure 6 highlights the key networks to illustrate the interconnectedness of metabolites within the biochemical pathways. The network visualization helped in identifying the clusters of highly correlated metabolites and their potential biological significance. The VIP metabolites and their correlations with each other such as p-cresol and phenol correlates with tyrosine and interlinked with dopamine, progesterone, estradiol, cortisone hormones, NADPH, NADP and oxygen molecule.

## Discussion

To align interpretation with measured shifts, we explicitly anchor pathways to the metabolites identified in results. Phenylalanine-tyrosine-tryptophan metabolism is linked to estrus – elevated phenol and p-cresol (Muniasamy et al. 2017); amino acid and nitrogen metabolism to glycine and ornithine (Doshi et al. 2024); glycolysis and TCA interfacing to glucose and alanine; antioxidant/ascorbate – aldarate metabolism to ascorbate (Li et al. 2024) and cholinergic/neuroendocrine signaling to o-acetylcholine (Wang et al. 2024). This comprehensive mapping reflects the coordinated endocrine-metabolic adaptation across estrus phases. Current estrus detection methods include visual, biochemical, and device-based approaches (e.g. vaginal biosensors, transrectal ultrasonography, activity monitors), though limitations like inflammation from vaginal probes persist (Fischer-Tenhagen et al. 2011; Verma et al. 2014). Advanced tools (e.g. BOVINOSE, ultra-wideband surveillance) improve detection efficiency (Wiegerinck et al. 2011). Few studies have explored the molecular basis of estrus using advanced techniques such as metabolomics (Hessock et al. 2023; Tribulo et al. 2019).

Urinary metabolites reflect phase-specific physiological changes during the estrus cycle, driven by hormonal regulation, metabolic demands (e.g. energy/protein balance), and oxidative stress management (Tan and Stowers 2020; Eom et al. 2021). Microbial activity also modulates hormone levels *via* microbiota β- glucuronidase – mediated estrogen deconjugation and immune – neuroendocrine signaling to the hypothalamus pituitary gonadal (HPG) axis, linking microbiota to estrus timing and behavior (Cotton et al. 2023; Zangirolamo et al. 2024). In bovines, reproductive tract microbiota vary with progesterone/estradiol across the cycle and during synchronization, supporting a bidirectional hormone – microbiome – behavior link relevant to estrus expression and fertility (Gupta et al. 2024). This provides a foundation for developing biomarkers for estrus detection, enhancing reproductive health monitoring, and refining management strategies in bovine breeding programs.

Compared to cattle and sheep, buffalo specific metabolomic studies of estrus cycle remain sparse. Table 3 summarizes metabolites linked to reproductive processes in livestock, emphasizing their biological roles. In this study, key urinary metabolites distinguishing estrus phases in buffaloes were identified: p-cresol and phenol (estrus); chlorogenate, o-acetylcholine and glycine (proestrus); ornithine (diestrus). Glycine, the most abundant amino acid in the oviduct and uterine fluids of cattle, also displays notable variation across reproductive stages in Awassi ewes (Ali and Abdulkareem 2025). The PCA loadings along with VIP scores from PLS-DA were used to identify key metabolites that differentiate the phases of estrus cycle in buffalo urine (Jolliffe 2005). These findings are consistent with PCA loadings and PLS-DA VIPs reported above.

**Table 3.** Urinary metabolites and their biological functions in the context of the estrus/reproductive cycle.

Metabolite	Origin	Relevance in context to estrus cycle/reproductive cycle	References
p-Cresol	Tyrosine Derivative	elicit behavioural changes during estrus (pheromone) in cow	(Vyas et al. 2012)
		indicate shifts in microbial activity in mouse	(Tan and Stowers 2020)
Glycine	amino acid	most abundant in cattle oviduct and uterine fluids	(Hugentobler et al. 2007)
		Awassi ewes across different reproductive stages	(Ali and Abdulkareem 2025)
Ornithine	urea cycle	identification of pregnancy-specific biomarkers in blood plasma of cattle after transfer of *in vitro* produced embryos in beef cattle	(Gómez et al. 2020)
Glucuronate	detoxification processes	metabolomics of blood, urine and ovarian follicular fluid at induced estrus stage in yak	(Zhao et al. 2024)
Glycocholate	a bile salt of lipid metabolism	Glycolate (classified as a lipid) were present in significantly higher concentrations than in the serum of dairy cow	(Eom et al. 2021)
Ascorbate	antioxidant	rabbits subjected to feed restriction showed higher levels of ascorbic acid, relate to oxidative stress or hormonal regulation	(Marín-García et al. 2024)
2-Hydroxyisobutyrate	ketogenic pathways	excreted at a lower concentration in urine of cows diagnosed with lameness post parturition using ¹ H-NMR	(Zhang et al. 2020)
o-Acetylcholine	a neurotransmitter precursor	clinical mastitis cow faces exhibit elevated o-acetylcholine, influencing neurological and reproductive processes during estrus	(Zhu et al. 2023)
5-Hydroxytryptophan (5-HTP)	amino acid	neurochemical regulation *via* serotonin secretion in Holstein steer	(Valente et al. 2021)
		signature urinary metabolites associated with early pregnancy in Mithun	(Sangwan et al. 2024)
3-Hydroxykynurenine	organic acid	reflect immune system activation or oxidative stress in dairy cattle	(Eom et al. 2021)
		signature urinary metabolites associated with early pregnancy in Mithun	(Sangwan et al. 2024)
Following metabolites have not been specifically reported for the reproductive cycle			
Chlorogenate	a phenolic antioxidant	linked to oxidative stress modulation and metabolic changes influenced by hormonal shifts in chicks	(Hu et al. 2021)
Glucose	carbohydrate	reflect metabolic demands or insulin regulation, usually present in very low amount in urine in bovine	(Foroutan et al. 2020)
3-Indoxylsulfate	an Indole	host-microbiome co-metabolite, concentration in urine reflects the microbial production of indole in the gut of humans and rats	(Banoglu and King 2002)
		metabolite identified in serum and urine of dairy cattle using ¹ H-NMR	(Eom et al. 2021)
3-methyl-2-oxovalerate	isoleucine catabolite, carboxylic acid	significantly higher in urine of cattle, (1-H-NMR) associated with ketosis diseases, inflammation, energy balance and body weight	(Eom et al. 2022)

Amino acid metabolism is very complex as large numbers of metabolites and multiple metabolic pathways are involved (Baker and Rutter 2023). Amino acids serve as a direct substrate for energy production and play a crucial role in glucose metabolism (Eom et al. 2021). Their utilization varies with shifts in carbohydrate and lipid metabolism, reflecting whole – body energy balance and substrate partitioning (Eom et al. 2021; Casaro et al. 2025). In the present study, several metabolites originating from amino acid metabolism were identified, consistent with recent livestock metabolomics studies (Doshi et al. 2024). o-Acetylcholine, a neurotransmitter precursor, increases during estrus in cows with clinical mastitis, suggesting its dual role in neurological and reproductive processes (Zhu et al. 2023). Glycine and ornithine, critical for protein metabolism and nitrogen balance, were elevated in proestrus. Glycine derivatives (N, N dimethylglycine and 2-furoglycine) modulate secretion of gonadotropins like FSH (Wang et al. 2013), while glycine itself – a major collagen protein (33% of amino acids) - also protects ovarian cells from oxidative stress (Li et al. 2018). Type VI collagen in the theca cell layers of follicles mediates interactions with extracellular matrix, influencing follicular cell dynamics (shape, proliferation, migration) during folliculogenesis (Iwahashi et al. 2000). Consistent with glycolysis-TCA coupling, elevated alanine levels during estrus in the present study support energy demands *via* gluconeogenesis and pyruvate conversion to acetyl-CoA for tricarboxylic acid (TCA) cycle, linking energy demand to estrus-associated glucose dynamics (Holeček 2024). Alanine and glycine in follicular fluid correlate positively with follicular growth (Matoba et al. 2014).

2-oxovalerate and 2-hydroxyisobutyrate, ketone bodies derived from valine/leucine catabolism (Jang et al. 2016) peaked during estrus (>6-folds) before declining diestrus. Though not directly linked to reproduction, their metabolic roles (e.g. mTOR signaling pathway *via* leucine catabolism) may directly support reproductive tissue growth (Laplante and Sabatini 2009). BCAA derived IGF-1 further activates kisspeptin through activation of PI3K/Akt/mTOR pathway in the hypothalamus upstream of GnRH (Mogami et al. 2009). Kisspeptin acts synergistically with LH and FSH to promote the expression of their receptors to promote follicular development (Harter et al. 2018).

Phenols (phenol, chlorogenate and p-cresol) – gut microbial tyrosine metabolites (Blachier 2023) – peaked during estrus in the present study. P-Cresol, a putative pheromone, induces bull flehmen behavior (Le Danvic et al. 2015; Muniasamy et al. 2017), and this correlates with ovulation in buffaloes and mares (Mozūraitis et al. 2012; Sudhan et al. 2017). Our findings show *a* > 5-fold rise in p-cresol/phenol during estrus, supporting their importance as urinary biomarkers.

Glucose supports metabolic demands during the follicular growth, ovulation, and luteal function. Cumulus cell glycolysis provides substrates for oocyte oxidative phosphorylation (Thompson et al. 2007), while LH surge – induced glucose byproducts in follicular fluid facilitate nutrient shuttling to oocytes and post-surge protein synthesis (Eppig et al. 2005; Gilbert et al. 2011). In anestrus cows, altered TCA cycle activity (elevated citric acid, reduced alanine/glutamate/asparagine) suggest metabolic imbalance (White 2015). Superior preovulatory follicles exhibit enriched glucose/TCA metabolites, correlating with oocyte competence and fertility (Read et al. 2022; Hessock et al. 2023).

Ascorbic acid (vitamin C), a key component of the ascorbate peroxidases-glutathione reductase (APXs-GR) antioxidant system, scavenges reactive oxygen species - superoxide (O_2_^•−^) and hydroxyl (^•^OH) radicals (Wang et al. 2017) and supports ovarian function by mitigating oxidative stress. It facilitates steroidogenesis, collagen synthesis, and follicular remodeling (Thomas et al. 2001). In follicular fluid, ascorbate sequestration promotes rapid follicular expansion and steroidogenesis in humans (Murray et al. 2001). Inside pituitary gland, ascorbic acid is involved in amidation of carboxy terminal of amino acids during synthesis of peptide hormones (Donlon and Ryan 2019).

O-acetylcholine, a cholinergic system component, modulates neuroendocrine pathways critical for reproductive hormone secretion and estrus cycle coordination. Cholinergic signaling influences the hypothalamic-pituitary-gonadal (HPG) axis, which regulates reproductive hormone release (Miller et al. 2004) and menstrual regularity (Veldhuis et al. 2010), highlighting its role in fertility (Picciotto et al. 2012). These interactions highlight the intricate crosstalk between neurotransmission and endocrine regulation (Figure 6) (Kolachi et al. 2025).

NMR based metabolomics, though non-destructive, quantitative, and highly reproducible, has lower sensitivity compared to mass spectrometry, which can limit the detection of low abundance metabolites in small urine volumes. Challenges such as dilution effects, baseline noise, and poor signal-to-noise ratios can also compromise quantification. NMR was selected here for a pilot discovery phase because it yields robust, standardized spectral fingerprints that are well suited for multivariate analyses in noninvasively collected urine samples. Combining NMR with mass spectrometry, along with refined data processing, and optimizing sample preparation, is expected to substantially strengthen robustness and translational utility.

This study identifies phase-specific metabolite expression patterns during the buffalo estrus cycle, visualized through heatmap clustering. However, the small sample size and single-site design limit the generalizability of the findings. Additionally, because all animals were from a single herd and geographical location, there is potential for site–specific or environmental bias. Future studies should include multi-site replication across diverse herds to strengthen generalizability of results. Future work should also expand cohorts, validate biomarkers for possible diagnostics development, pending external validation and incorporate longitudinal assessments of nutritional and environmental impacts on metabolic profiles.

## Conclusion and future prospective

This study provides a comprehensive report on differential urinary metabolite profile in buffalo during pro-estrus, estrus and diestrus using ^1^*H* NMR analysis and elucidates the involvement of relevant metabolic pathways. The results indicate phase specific abundance of urinary metabolites – most notably p-cresol and phenol for estrus, chlorogenate and o–acetylcholine for proestrus, and ornithine for diestrus – that may serve as candidate biomarkers to support accurate estrus detection in buffalo, pending external validation. These findings offer a foundation for developing field – oriented kits and sensors for estrus detection and for refining breeding management, while recognizing that small sample size and single-site design necessitate confirmation in larger, multi-site cohorts and independent test sets to establish generalizability and robustness. Future work will integrate complementary MS based platforms to expand metabolite coverage and sensitivity, undertake targeted validation of priority markers, and evaluate performance under diverse herd, nutritional, and environmental conditions to facilitate translational application.

## Supplementary Material

Supplementary File.docx

## Data Availability

The data that support the findings of this study are available from the ICAR-Central Institute for Research on Buffaloes, Hisar through the corresponding author, Ashok K. Balhara, upon reasonable request.
