# Supplementary material for: Urinary metabolite signatures as predictive biomarkers for estrus detection in water buffaloes: a proton-NMR based study
Source: Vet Q. 2025 Dec 13;45(1):2593361. doi: 10.1080/01652176.2025.2593361 (PMC12915391; doi:10.1080/01652176.2025.2593361)
Supplement: Supplementary File.docx [file TVEQ_A_2593361_SM8334.docx]

**
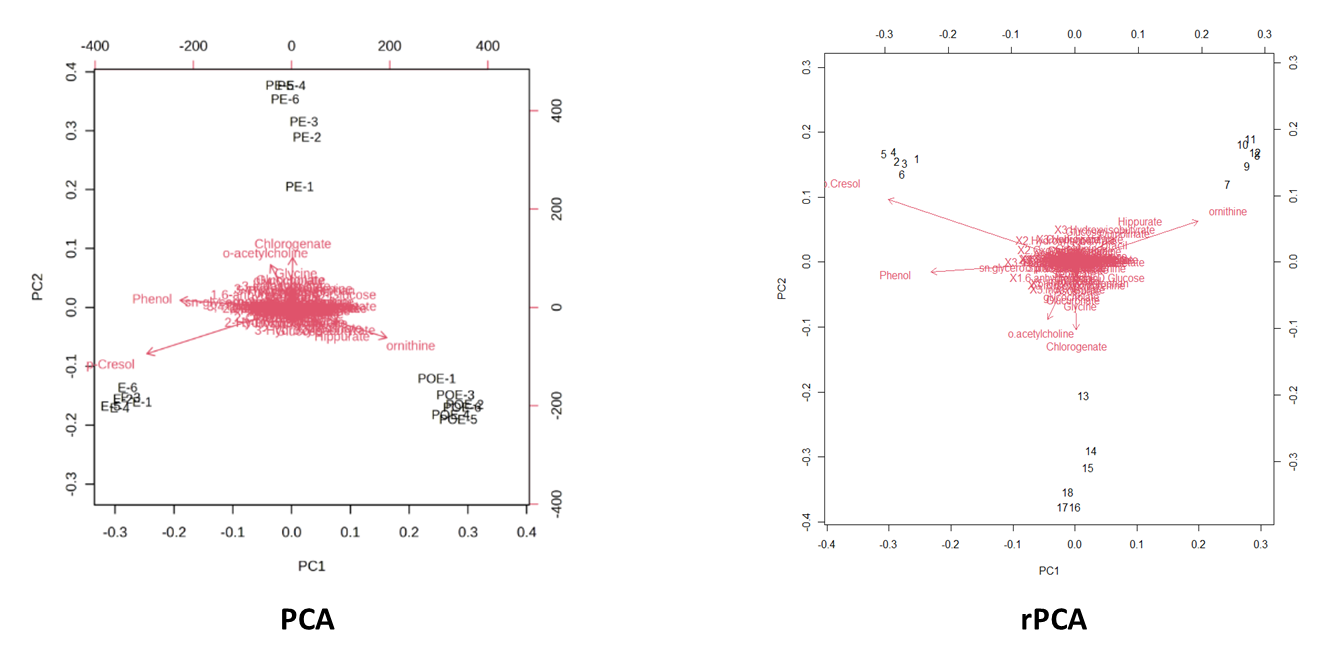
**

**Supplementary Figure 1.** PCA and rPCA biplots for the distinction in oestrous phases

**
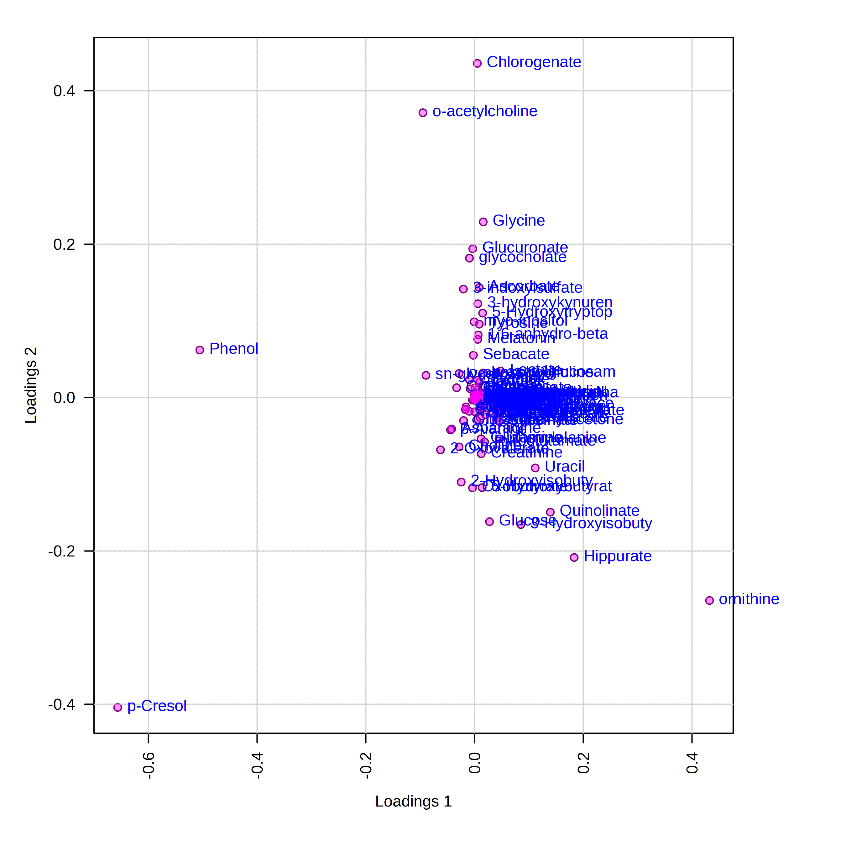
**

**Supplementary Figure 2.** Loadings plot for PCA recognized p-cresol, ornithine, phenol, chlorogenate, o-acetylcholine, Hippurate and glycine as the prominent metabolites responsible for differentiation

**
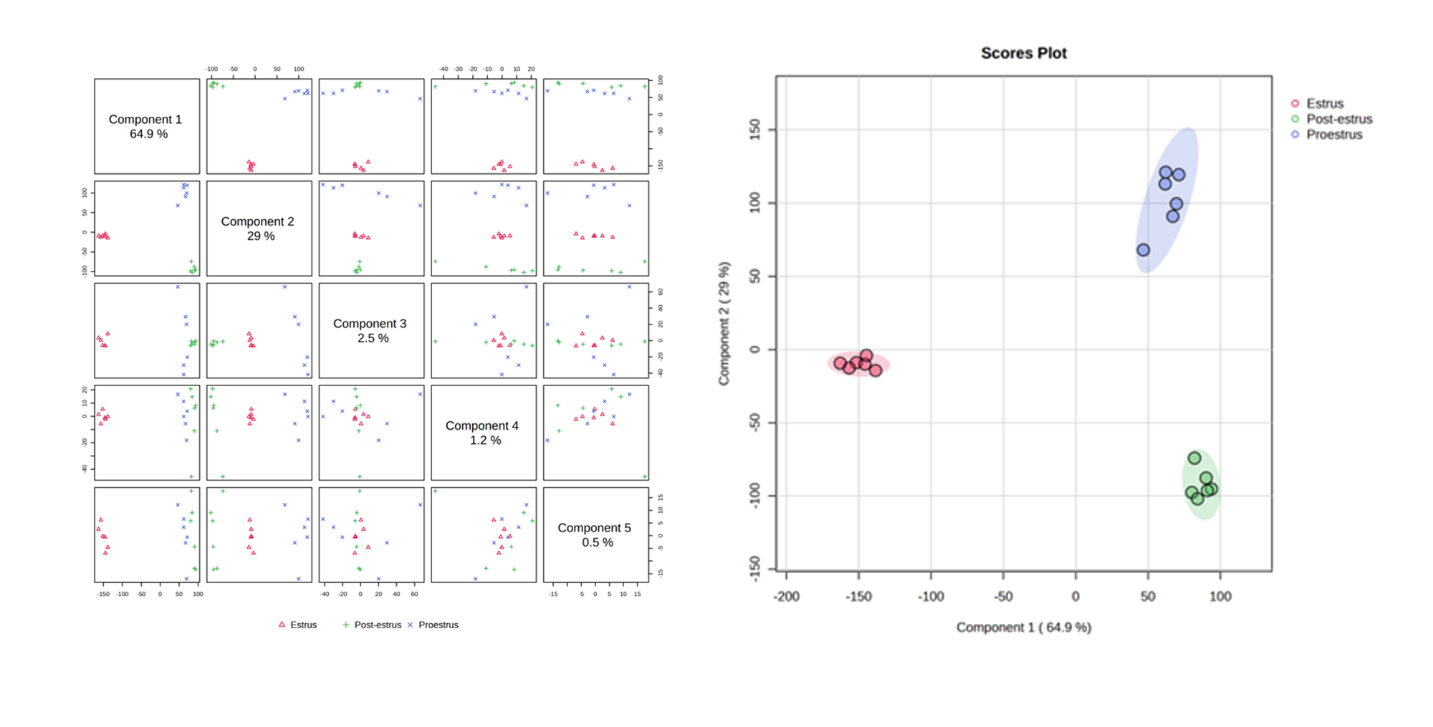
**

**Supplementary Figure 3.** (a) PLS-DA showing variability explained by PC1 (64.9%), PC2 (29%) and PC3 (2.5%). The 2D score plot of PLS-DA analysis incorporating component 1(64.9%) and component 2 (29%) depicting prominent clusters of metabolomic data of estrus (red), pro-estrus (blue) and post-estrus (green) phases of the estrous cycle. (b) The scores plot showing distinct clustering of buffalo urine metabolomic profiles during different estrus phases. Each point represents an individual sample, with coloured ellipses indicating 95% confidence regions for each oestrus phase.
